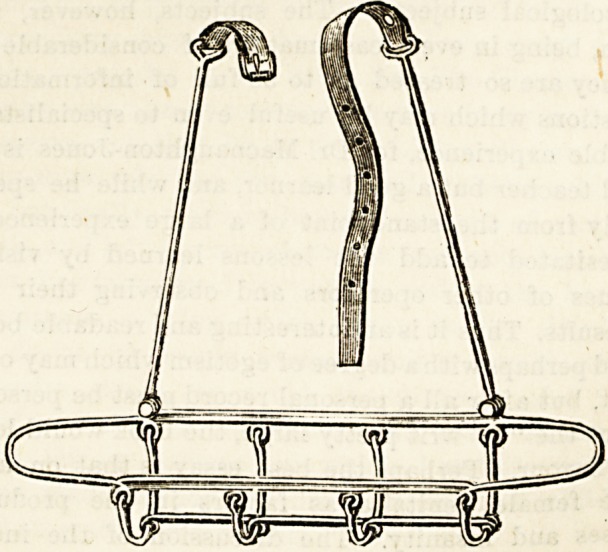# New Appliances and Things Medical

**Published:** 1903-02-07

**Authors:** 


### NEW APPLIANCES AND THINGS MEDICAL.

### SANITARY WIRE ARM SLING.

(Agent: J. Panton Ham, 32 Essex Street, Strand, W.C.)

These new arm slings, patents for which are under application, possess many obvious advantages. They are strong, light, and extremely sanitary, and at the same time they are sufficiently flexible to enable them to be moulded to the shape of the arm in such a way as to afford support in the positions required. They can be easily applied and adjusted, and again taken off without trouble or inconvenience to the patient. We strongly commend them to the notice of surgeons.

### ANTISEPTIC AND SANITARY HEALING POWDER.

(Nurse May, Oxford House, Clevedon, Somerset.)

The above is a good sample of an antiseptic starch powder for toilet and nursery use. It contains an antiseptic which is universally regarded as one of the best for dermatological use. It is soothing and healing to the skin.

## Figures and Tables

**Figure f1:**
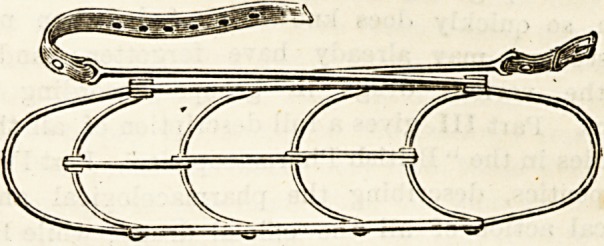


**Figure f2:**
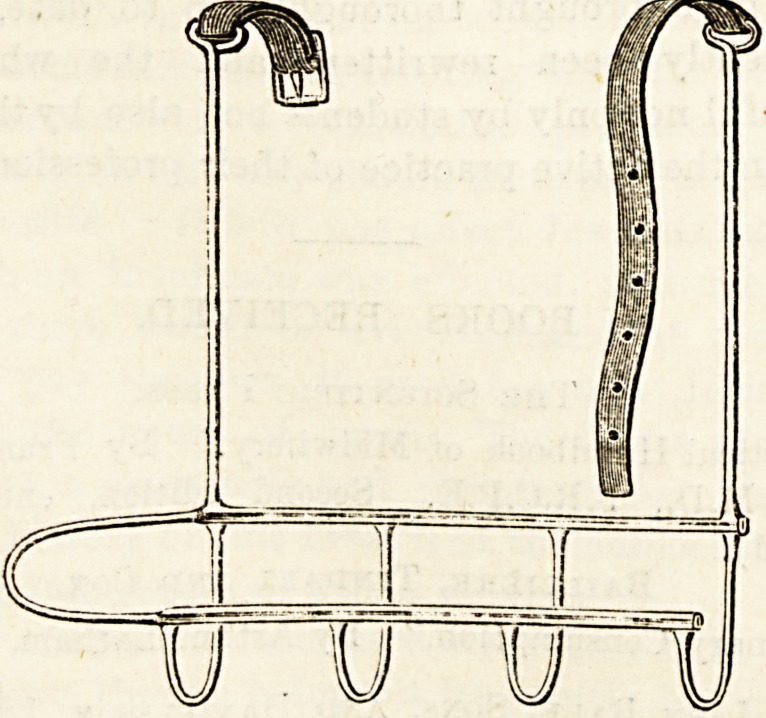


**Figure f3:**